# Field energetics and lung function in wild bottlenose dolphins, *Tursiops truncatus*, in Sarasota Bay Florida

**DOI:** 10.1098/rsos.171280

**Published:** 2018-01-17

**Authors:** A. Fahlman, M. Brodsky, R. Wells, K. McHugh, J. Allen, A. Barleycorn, J. C. Sweeney, D. Fauquier, M. Moore

**Affiliations:** 1 Fundación Oceanografic de la Comunidad Valenciana, Gran Vía Marques del Turia 19, 46005 Valencia, Spain; 2 Texas A&M University-Corpus Christi, 6300 Ocean Drive, Corpus Christi, TX 78412, USA; 3 Woods Hole Oceanographic Institution, 266 Woods Hole Rd., MS# 50, Woods Hole, MA 02543-1050, USA; 4 Micah Brodsky, V.M.D. Consulting, Miami Shores, FL 33138, USA; 5 Chicago Zoological Society's Sarasota Dolphin Research Program, c/o Mote Marine Laboratory, 1600 Ken Thompson Parkway, Sarasota, FL 34236, USA; 6 Dolphin Quest, Oahu, 5000 Kahala Ave, Honolulu, HI 96816, USA; 7 Marine Mammal Health and Stranding Response Program, Office of Protected Resources, NOAA/National Marine Fisheries Service, 1315 East-West Highway, Room 13620, Silver Spring, MD 20910, USA

**Keywords:** field metabolic rate, pulmonary function test, tidal volume, diving physiology, marine mammals, spirometry

## Abstract

We measured respiratory flow rates, and expired O_2_ in 32 (2–34 years, body mass [*M*_b_] range: 73–291 kg) common bottlenose dolphins (*Tursiops truncatus*) during voluntary breaths on land or in water (between 2014 and 2017). The data were used to measure the resting O_2_ consumption rate ($\dot{\text{V}}{\text{O}}_{2}$, range: 0.76–9.45 ml O_2 _min^−1 ^kg^−1^) and tidal volume (*V*_T_, range: 2.2–10.4 l) during rest. For adult dolphins, the resting *V*_T_, but not $\dot{\text{V}}{\text{O}}_{2}$, correlated with body mass (*M*_b_, range: 141–291 kg) with an allometric mass-exponent of 0.41. These data suggest that the mass-specific *V*_T_ of larger dolphins decreases considerably more than that of terrestrial mammals (mass-exponent: 1.03). The average resting $\text{s}\dot{\text{V}}{\text{O}}_{2}$ was similar to previously published metabolic measurements from the same species. Our data indicate that the resting metabolic rate for a 150 kg dolphin would be 3.9 ml O_2 _min^−1^ kg^−1^, and the metabolic rate for active animals, assuming a multiplier of 3–6, would range from 11.7 to 23.4 ml O_2 _min^−1^ kg^−1^.\absbreak Our measurements provide novel data for resting energy use and respiratory physiology in wild cetaceans, which may have significant value for conservation efforts and for understanding the bioenergetic requirements of this species.

## 1. Introduction

Climate change is causing perturbations in oceanic and coastal circulation and water temperature, which can alter the spatial and temporal distribution of animals and their food resources [1]. Increased utilization of the planet's oceans by a growing human population has impacted marine ecosystems. For marine mammals known effects include increased ocean noise, increased incidence of human interactions like fishing gear entanglements and ship strikes, exposure to toxic chemicals (e.g. organochlorines such as DDT, PCBs, heavy metals, etc.), and eutrophic waste from agriculture, expanding industry and developing urban areas. Previous studies have suggested that marine predators may be valuable bio-indicators of environmental health [2]. As top-level predators, marine mammals may be particularly prone to effects of climate change and deteriorating environmental conditions; serving as the proverbial canary in the coal-mine, indicating significant changes at lower trophic levels and showing the impact those changes could have on other mammalian species, including humans, that share the same water, air and fish.

An essential aspect of understanding the ecology and distribution of any species is to define their resource and energy requirements. While considerable work has been done on the bioenergetics and respiratory physiology of seals and sea lions, technical and logistical limitations have prevented comparable measurements in wild cetaceans. One approach to estimate field metabolic rate in large whales has been to measure their breathing frequency [3–8]. The assumption is that increased breathing, as occurs during active foraging or travel, is tightly linked to the energy requirements of the activity state and can be used to assess the field metabolic rate for different activities, e.g. resting or swimming at the surface and diving. While this method is attractive in its simplicity, it assumes that the average tidal volume (*V*_T_) and O_2_ exchange fractions are known, and that they remain constant over the measurement period. This is not always the case [9], and improved knowledge of the cardiorespiratory physiology, and the temporal patterns of respiration and gas exchange following exercise or diving would significantly improve the estimate of metabolic cost [10–12]. There are also other techniques that can provide proxy estimates of field metabolic rate, such as the dilution of doubly labelled water in the body water pool, activity records or heart rate monitoring, but these techniques require careful validation to assess how changes in their relationships may change over different seasons, years, activities, life stage, etc. Still, the resting metabolic rate (RMR) is often used as a tool to provide an estimate of the minimum energy requirements for a species.

Our objective was to measure lung function and estimate RMR for common bottlenose dolphins (*Tursiops truncatus*), using breath-by-breath respirometry in wild animals. Understanding metabolic and respiratory requirements is of particular interest because it is vital to identifying and understanding the physiological limitations for survival of a species. In addition, respiratory disease is a major cause for morbidity and mortality in wild cetaceans, and increasing environmental stressors, such as exposure to pollutants, is likely to exacerbate this problem [13,14]. Despite significant advances in technology, we still know very little about the respiratory physiology of marine mammals. Because respiratory physiology is difficult to study in free-ranging dolphins, normal lung function data for wild animals are limited [15,16]. We therefore compared data collected from wild animals with data from healthy dolphins and cetaceans managed under human care [17–21] to assess if respiratory physiology measurements may provide information that is relevant to both populations.

## 2. Material and methods

### 2.1. Animals

The free-ranging bottlenose dolphins were long-term residents of Sarasota Bay (Lat: 27°22′40.31^″^ N Long: 82°35′9.40^″^ W) and were sampled during 5–9 May 2014; 12–20 May 2015; 6–11 May 2016 and 8–12 May 2017. Juvenile and adult bottlenose dolphins of both sexes, and varying sizes (table 1) were used for the experiments. These animals were measured during periodic capture-release health assessments by the Sarasota Dolphin Research Program (SDRP), and were briefly encircled with a net, examined and sampled before being released on site [2]. All spirometry trials (breath-by-breath lung function and end-expired O_2_) were measured from voluntary breaths while the dolphin laid on a shaded, padded mat on the deck of a boat or was gently restrained while partially submerged in water. All work was approved by the IACUC at Texas A&M University Corpus Christi (TAMUCC-IACUC AUP#04–11) and by a research permit issued by the National Marine Fisheries Service (Scientific Research Permit No. 15543).

**Table 1.** Year tested, animal identification (freeze-brand number), sex (M-male, F-female), body mass (*M*_b_), straight length (SL), maximum girth (G), approximate age, breathing frequency and (*N*) number of breaths collected while in air or in water. Symbols ‘—’ indicate no data available.

							*N*		*f*_R_ (breaths min^−1^)	
year	animal ID	sex	*M*_b_ (kg)	length (cm)	girth (cm)	age (years)	air	water	air	water
2014	FB196	M	248	269	152	16	63	8	2.5	2.7
	FB268	M	227	273	147	21	72	—	2.8	1.5
	FB276	M	291	285	163	22	75	12	3.7	1.0
	FB142	M	256	274	153	22	55	14	2.7	1.8
	FB237	F	192	254	142	32	104	17	2.2	3.6
	FB239	F	117	228	113	4	—	11	2.2	2.4
	FB197	F	169	234	141	11	51	—	1.9	2.1
	FB241	F	157	242	137	7	—	28	1.3	1.1
	FB175	F	170	249	137	23	36	—	4.3	3.8
	FB133	F	167	242	138	15	60	17	2.6	2.2
	FB242	M	276	281	159	24	28	11	0.9	1.1
	FB164	M	291	262	165	25	47	16	0.8	1.9
	FB185	F	172	252	130	14	82^a^	17^a^	3.8	4.2
2015	FB251	F	146	240	127	>12	17	—	1.4	—
	FB245	F	110	212	114	3	104	37	3.6	4.9
	FB133	F	165	240	135	16	303	60	4.5	4.5
	FB199	F	142	236	125	13	27	25	4.8	5.6
	FB257	F	73	195	103	2	—	41	—	3.4
	FB193	F	155	250	126	31	—	19	—	3.2
	FB146	M	266	277	153	19	70	42	1.5	4.1
	FB254	M	250	273	156	26	34	10	4.0	1.6
2016	FB209	F	154	236	135	12	102	12	2.6	—
	FB255	F	81	192	108	3	15	22	2.4	1.3
	FB33	F	195	258	142	34	223	13	2.8	—
	FB259	F	100	216	112	3	1	17	—	1.2
	FB292	M	124	216	124	4	84	15	3.2	4.6
	FB294	M	95	202	114	3	46	9	4.7	1.2
	FB223	F	160	251	131	15	8	17	2.3	2.3
	FB178	M	248	272	149	21	63	19	3.7	—
	FB188	M	229	257	150	20	56	16	3.0	—
2017	FB296	M	136	228	124	4	60	61	3.3	6.5
	FB306	M	101	208	115	4	49	53	3.2	5.8

^a^Gas analyser not working.

### 2.2. Respiratory flows (lung function)

The procedures and equipment used were identical to those used in our previous study on the same species under human care [19], which are briefly summarized below. Respiratory flows were measured using a custom-made Fleisch type pneumotachometer (Mellow Design, Valencia, Spain; or Micah Brodsky, V.M.D. Consulting, Miami, FL, see fig. 1 in [19]), using a low-resistance laminar flow matrix (Item # Z9A887–2, Merriam Process Technologies, Cleveland, OH). A differential pressure transducer (Spirometer Pod, ML 311, ADInstruments, Colorado Springs, CO) was connected to the pneumotachometer with two, 310 cm lengths of 2 mm I.D., firm-walled, flexible tubing. The differential pressure transducer was connected to a data acquisition system (Powerlab 8/35, ADInstruments, Colorado Springs, CO), and the data were captured at 400 Hz and displayed on a laptop computer running LabChart (v. 8.1, ADInstruments, Colorado Springs, CO). The differential pressure was used to determine flow and was calibrated using a 7.0 l calibration syringe (Series 4900, Hans-Rudolph Inc., Shawnee, KS). The signal was integrated and the flow determined as detailed previously [19].

### 2.3. Respiratory gas composition

The concentration of expired O_2_ was subsampled via a port in the pneumotachometer and passed through a 310 cm length of 2 mm I.D., firm-walled, flexible tubing and a 30 cm length of 1.5 mm I.D. Nafion tubing, to a fast-response O_2_ and CO_2_ analyser (Season 2016–2017: Gemini respiratory monitor, CWE Inc.; Season 2014–2015: ML206, Harvard Apparatus, Holliston, MA, USA) at a flow rate of 200 ml min^−1^. However, the CO_2_ analyser was only working during the experiments in 2016 and we therefore do not report these data. The gas analyser was connected to the data acquisition system and sampled at 400 Hz. The gas analyser was calibrated before and after the experiment using a commercial mixture of 5% O_2_, 5% CO_2_ and 90% N_2_ (Product No. 17 L-340, GASCO, Oldsmar, FL). Ambient air was used to check the calibration before and after each experimental trial. Mean air temperature and humidity during trials were 28.2 ± 3.3°C (*n *= 74, range 22.6–35.4°C) and 75.3 ± 17.5% (44–99%). The average (±s.e., *n *= 19) surface water temperature around the sampling boat was 26.5 ± 0.5°C (range: 22.7–29.7°C).

### 2.4. Metabolic rates

The metabolic rates were estimated as previously detailed [19], and the methods are briefly summarized here. The respiratory gas signals were phase-corrected for O_2_, to match the respirations, and the expiratory flow rate and expired O_2_ content were multiplied to calculate the instantaneous volume of oxygen rate ($\dot{\text{V}}{\text{O}}_{2}$). The instantaneous $\dot{\text{V}}{\text{O}}_{2}$ was integrated over each breath to yield the total volume of O_2_ exchanged during each breath. The O_2_ volumes were summed for each trial period and divided by the duration of the trial to provide an estimate of the oxygen consumption rate for that time period. All dolphins were calm for at least 20 min before sampling commenced. Sampling for respiratory flow and expired gas content continued during most of the duration on the deck and up to 15 min in water. Data were selected to include sections with continuous measurements of at least 3 min. For example, if there were concerns that the flow meter did not seal correctly around the blow-hole, the breath was excluded and the duration started over. Thus, while 3 min may be a shorter duration to collect data than some studies, the total duration of measurement was considerably longer, ranging from 20 to 60 min.

### 2.5. Data processing and statistical analysis

All gas volumes were converted to standard temperature pressure dry (STPD, [22]). Exhaled air was assumed saturated at 37°C, inhaled air volume was corrected for ambient temperature and relative humidity.

Metabolic data are reported as the average O_2_ consumption rate for an entire trial. The relationship between a dependent variable and experimental covariates was analysed using linear-mixed effects models (lme, R: A Language and Environment for Statistical Computing, R Foundation for Statistical Computing, version 3.1.0, 2014). The individual animal was treated as a random effect, which accounted for the correlation between repeated measurements on the same individual [23]. Initially, a univariate analysis was used to determine which variables to consider in a multivariate model. Variables with a *p* < 0.2, using the Wald test and log-likelihood ratio test, were considered in a multivariate model. We used the log-likelihood ratio test and the Akaike information criterion (AIC) to determine which parameters warranted inclusion in a nested model. In this study, *p*-values *p* ≤ 0.05 and *p* < 0.01 were considered significant and highly significant, respectively, and *p* ≤ 0.1 was considered a trend. Data are presented as the mean ± standard deviation (s.d.), unless otherwise stated.

Initially we analysed all age groups together, but we also separated animals into juveniles (less than 10 years) and adults (greater than or equal to 10 years) in order to compare allometric changes for the entire dataset and for each age class separately.

## 3. Results

Data from 2475 spontaneous breaths were collected over 4 years (2014–2017) from 32 male and female juvenile and adult bottlenose dolphins (table 1), living in and around Sarasota Bay, Florida. Measurements on the deck occurred over 20–60 min, and in water 15–20 min, with continuous measurements for at least 3 min.

### 3.1. Respiratory flows and timing

The average respiratory frequency (*f*_R_) during trials was 2.7 ± 1.3 breaths min^−1^ (range: 0.8–6.5), and did not differ among years, sex, with body mass (*M*_b_) or position (whether held in or out of the water; all *p *> 0.1, *t*-value: 0.8).

**Figure 1.**
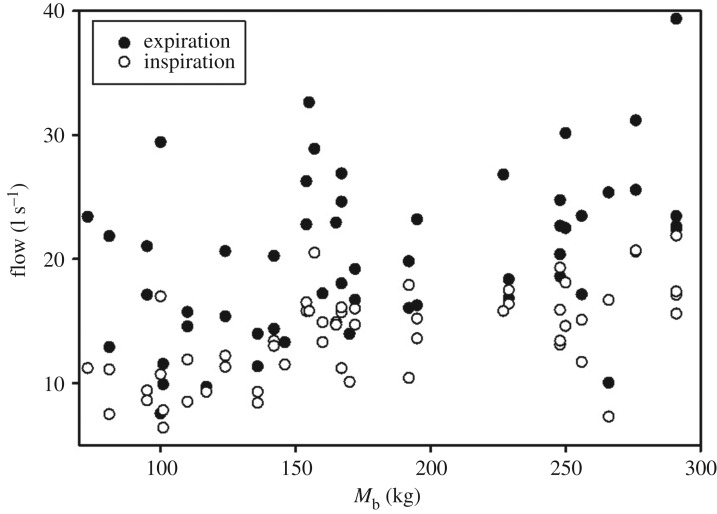
Expiratory or inspiratory respiratory flow (l s^−1^) versus body mass (*M*_b_, kg) for dolphin either in air or in water.

The average maximum inspiratory flow of spontaneous breaths was 13.8 ± 3.8 l s^−1^ (range: 6.4–21.9 l s^−1^), which was significantly lower than the maximum spontaneous expiratory flow of 20.0 ± 7.5 l s^−1^ (range: 7.5–39.4 l s^−1^, *t*-ratio = 10.9, *p* < 0.01, paired-*t* test). The log_10_-transformed inspiratory (Inspflow, χ^2 ^= 20.6, 1 d.f., *p* < 0.01) and expiratory flow (Expflow, *χ*^2 ^= 14.2, 1 d.f., *p* < 0.01) correlated with log_10_-transformed *M*_b_ (log_10_ [*M*_b_], figure 1), but neither sex nor position (in-water versus out of water) affected inspiratory or expiratory flow. When separating animals into juveniles and adults, *M*_b_ did not correlate with respiratory flow (*χ*^2 ^= 2.9, 1 d.f., *p* > 0.05).

**Figure 2.**
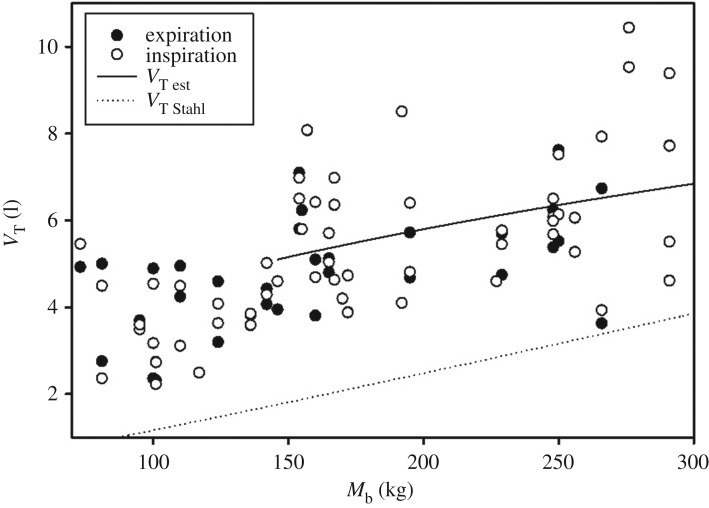
Inspiratory tidal volume (*V*_T_, l) versus body mass (*M*_b_, kg) for dolphins either in air or in water. Solid line is the estimated *V*_T_ for adult dolphins (equation (3.1)) and the dotted line the regression equation for terrestrial mammals [24].

There were no differences in inspiratory (5.3 ± 1.8 l, range: 2.2–10.4) or expiratory (5.2 ± 1.8 l, range: 2.3–10.4) *V*_T_ (*p *> 0.05, *t*-stat: 1.9). For all age groups, only *M*_b_ correlated with *V*_T_ (*χ*^2 ^= 22.8, 1 d.f., *p* < 0.01, figure 2). When separating animals into juveniles (less than 10 years) and adults (greater than or equal to 10 years), log_10_ [*M*_b_] warranted inclusion for adults (*p *< 0.05, *χ*^2 ^= 5.2, 1 d.f.):

3.1 $$\text{lo}{\text{g}}_{10}[V_{T}]=-0.18\;(\pm 0.22)+0.41\;(\pm 0.17)\cdot \text{lo}{\text{g}}_{10}[M_{b}],$$

The duration of the expiratory phase (0.437 ± 0.136 s) was significantly shorter than that of the inspiratory phase (0.512 ± 0.112 s), and neither inspiratory nor expiratory durations changed with sex, *M*_b_, or whether animals were in water or out of water (*p *> 0.1 for all).

### 3.2. Gas exchange

The average (*n* = 56) end-expiratory O_2_ was 10.4 ± 1.4%, and was significantly higher in female dolphins (10.8 ± 1.4%, *n *= 35) as compared with males (10.0 ± 1.4%, *n *= 21, *p* < 0.05, Wald test). In addition, there was a trend that females or being positioned in the water had higher end-expired O_2_ compared with males or animals positioned out of water (*χ*^2 ^= 3.08, 1 d.f., *p* < 0.08).

### 3.3. Metabolic rates

**Figure 3.**
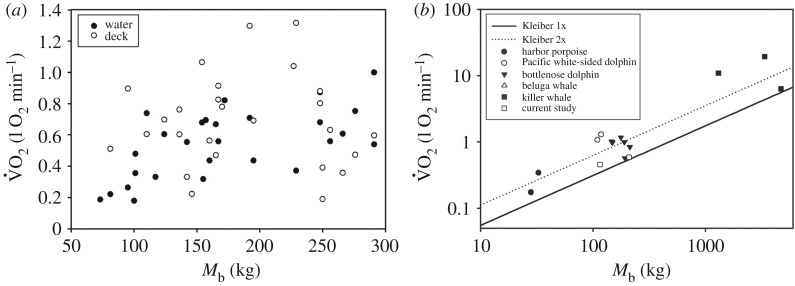
Rate of O_2_ consumption ($\dot{\text{V}}{\text{O}}_{2}$, l O_2 _min^−1^) versus body mass (*M*_b_, kg) for (*a*) dolphins in air and in water from the current study. (*b*) For a range of cetaceans from the following studies: harbour porpoise [25,26], Pacific white-sided dolphin [27,28], bottlenose dolphin [17–20], beluga whale [29], killer whale [30,31].

Resting $\dot{\text{V}}{\text{O}}_{2}$ (RMR) was estimated for periods during which the dolphin had remained calm for a minimum of 20 min. The sample duration used for calculating the $\dot{\text{V}}{\text{O}}_{2}$ ranged from 3 to 15 min (figure 3*a*). The estimated RMR varied substantially within and among animals and the mass-specific $\dot{\text{V}}{\text{O}}_{2}$ ($\text{s}\dot{\text{V}}{\text{O}}_{2}$) ranged from 0.76 ml min^−1 ^kg^−1^ to 9.45 ml O_2 _min^−1^ kg^−1^ (figure 3*a*). There was a correlation between log_10_-transformed $\dot{\text{V}}{\text{O}}_{2}$ (log_10_[$\dot{\text{V}}{\text{O}}_{2}$]), *M*_b_ (log_10_[*M*_b_]) and whether animals were positioned in (position = 1) or out of the water (position = 0, figure 3*a*, *p* < 0.05, *χ*^2 ^= 5.7, 1 d.f.)

3.2 $$\text{lo}{\text{g}}_{10}[\dot{\text{V}}{\text{O}}_{2}]=-1.15\;(\pm 0.40)+0.43\;(\pm 0.18)\cdot \text{lo}{\text{g}}_{10}[M_{b}]-0.13\;(\pm 0.05)\;\cdot \text{position.}$$

but neither year nor sex were significant.

When separating animals into juveniles and adults, both position and log_10_[*M*_b_] warranted inclusion for juveniles (*p* < 0.01, *χ*^2 ^= 9.0, 1 d.f.)

3.3 $$\text{lo}{\text{g}}_{10}[\dot{\text{V}}{\text{O}}_{2}]=-2.75\;(\pm 0.71)+1.25\;(\pm 0.35)\;\cdot \text{lo}{\text{g}}_{10}[M_{b}]-0.23\;(\pm 0.08)\cdot \;\text{position.}$$

but neither position or *M*_b_ warranted inclusion for adults (*p *> 0.1, *χ*^2 ^= 2.1, 1 d.f.).

## 4. Discussion

We estimated RMR and measured lung function parameters across 2 age classes of bottlenose dolphins (juvenile and adult) over a 4-year period. When both age groups were analysed together, RMR and *V*_T_ rate scaled with *M*_b_. When the groups were separated into juveniles and adults, RMR only correlated with *M*_b_ for juveniles. *V*_T_ scaled with *M*_b_ for adults with an allometric mass-exponent of 0.41 ± 0.17. These results are similar to those measured in bottlenose dolphins under human care, suggesting that data from the latter group provide physiological information of relevance for wild dolphins.

The measured *V*_T_ collected in this study agree well with previous measurements in cetaceans [16]. The average mass-specific *V*_T_ (s*V*_T_) recorded in the current study was 30.9 ml kg^−1^ (range: 13.6–67.5 ml kg^−1^), which is similar to previous data for the bottlenose dolphin (27.5–58.8 ml kg^−1^, [9,19,32]), the harbour porpoise (39.3–52.6 ml kg^−1^ in [25]) and grey whale (12.0–35.3 ml kg^−1^, [33]). Results from these past studies are from a range of experiments with animals positioned on land or in water, and from voluntarily participating animals in human care to restrained free-ranging animals. For all data, both the respiratory flow and *V*_T_ scaled with *M*_b_, but when separated into juveniles and adults, only *V*_T_ from the latter group correlated with size. Unlike terrestrial mammals, where the mass-exponent is close to 1 (table 1 in [24]), *V*_T_ scaled allometrically with a mass-exponent of 0.41 (equation (3.1)). Consequently, the s*V*_T_ decreased with *M*_b_ and for a 150 kg and a 300 kg dolphin was 34 ml kg^−1^ and 23 ml kg^−1^, respectively. Similarly, the *V*_T_ as a percentage of the estimated total lung capacity (TLC_est_) decreased from 38% for a 150 kg dolphin, to 27% for a 300 kg animal [16,34]. The *V*_T_ in the dolphin was greater when compared with terrestrial mammals of comparable size, but the relative difference decreased with size.

The breathing strategy of adult terrestrial mammals involves a high respiration frequency *f*_R_, low *V*_T_, and a brief respiratory pause following expiration. Aquatic mammals, on the other hand, have a lower *f*_R_, a greater *V*_T_ and a respiratory pause that often lasts for several seconds to minutes following inhalation [16,35]. It has been hypothesized that the aquatic breathing strategy may enhance buoyancy management [35], or help keep the arterial partial pressure of CO_2_ (PACO_2_) at levels similar to that of land mammals [36]. Minute ventilation, the product of *V*_T_ and *f*_R_, is regulated to deliver O_2_ to and remove CO_2_ from the alveoli. In terrestrial mammals, *V*_T_ (1.04) scales isometrically while *f*_R_ (−0.26) is inversely related to *M*_b_, resulting in a mass-exponent for minute volume that scales with the metabolic rate [24]. Thus, the allometric changes in minute ventilation in terrestrial mammals are governed by an *f*_R_ that decreases with *M*_b_. The data reported in the current study suggest that the decrease in mass-specific minute ventilation with size is governed by a reduction in *V*_T_, while *f*_R_ seems to remain constant. This agrees with other data reported for cetaceans, where the mass-exponent for *f*_R_ was not different from 0 (see data on cetaceans in table 1 in [35]). Thus, as mass-specific RMR decreases with size, the reduction in minute ventilation is caused by a reduction in mass-specific *V*_T_. Variation in *V*_T_ is more efficient to alter alveolar ventilation as it reduces the dead space ventilation. Consequently, the allometric changes in cetaceans may be an evolutionary consequence that helps reduce the work of breathing and enhances gas exchange in the aquatic environment.

The basal metabolic rate (BMR) of an animal is an estimate of the energetic cost of the basic functions required to sustain life, and it is measured under highly specific and controlled conditions, e.g. adult animals in a thermoneutral environment. In field situations, it is logistically challenging to meet these conditions, and RMR often serves as a proxy for BMR. Relatively few measurements of RMR have been made in marine mammals, and most of these have been made on animals in managed care or with animals held under quasi-natural conditions [17–21,25,37–43]. Many of the older studies concluded that the mass-specific RMR is considerably higher in aquatic species than in their terrestrial counterparts. Possible explanations for the higher RMR in dolphins include increased energetic demands due to thermoregulation or a high protein diet [44–47]. More recent studies, where the sample techniques have been refined for marine mammals, have measured RMRs that are at or near basal levels [18,21,27,29,30]. Thus, there is currently considerable controversy whether estimated BMR/RMR in marine mammals is truly elevated as compared to terrestrial species, and criticisms include limitations with experimental design (e.g. duration of measurements) and analysis of collected data [48–50]. Studies have shown that variation in RMR is affected by body condition, the thermal environment, desensitization through training and psychological state [18,19,29,30,51,52]. These past studies indicate that experimental design and conditions may significantly alter the results and could explain the large variability in RMR reported across published studies. In past studies, the mass-specific RMR for bottlenose dolphins in water ranged from 3.0 to 7.0 ml O_2 _min^−1^ kg^−1^ (*M*_b_ range 125–250 kg, [10,17–20,53]), and were in the same range as the values reported in the current study (figure 3*b*).

In the current study, the data from all age groups indicate that RMR scaled allometrically with a mass-exponent that was significantly lower than those reported for terrestrial mammals (equation (3.2), [54,55]. In land mammals, BMR in adults is well known to scale allometrically with body size over several orders of magnitude, but may vary greatly within species or a narrow weight range. For example, the mass-specific value may vary 2- to 3-fold, and the variation explained by *M*_b_ is often low [56,57]. When the data were separated into juveniles and adults, RMR only correlated with *M*_b_ for the juveniles (figure 3*a*). The mass-exponent for juveniles was greater than the adult value in terrestrial mammals (equation (3.3)).

There are a number of possible reasons for our findings. First, the measurements in the current study do not fulfil the criteria for BMR; we studied juvenile and adult animals and had no control over whether the animals were pre- or post-prandial. This may increase the variation, but we should also expect the mass-specific RMR to be higher as younger animals tend to have greater energetic requirements as they allocate considerable energy for growth. Second, breath-by-breath respirometry was used in the current study, which is experimentally challenging [19]. These limitations have been discussed previously, and it is unlikely that limitations in the experimental design would have significantly affected the results. Factors such as stress and personality are known to alter metabolic rate, and while stress often causes hypermetabolism, some species respond with a reduction in metabolic rate [56,58,59]. This may explain the low RMR in the current study, and wild dolphins may respond by reducing metabolic rate in response to any stress associated with capture. Fourth, in the current study the dolphins had been restrained for at least 20 min before measurements began, followed by repeated measurements ranging from 3 to 15 min. In some previous studies, the resting period ranged from 40 s to 4.5 min [17,19,20]. As the duration of measurement of RMR is important [21,27,60], it is possible that these extended measurement periods helped calm the animals and reduce the RMR. In fact, studies where the dolphins were resting for 10–20 min in the respirometer have reported values that are similar to those predicted by Kleiber [18,21,29,52]. Consequently, appropriately designed metabolic studies on marine mammals managed under human care provide RMR values that are similar to those in wild populations.

Studies indicate that RMR is about 30–40% of daily energy requirement [61,62], and understanding a species' metabolic cost is important for assessing energy flow within populations and ecosystems. For marine mammals, estimated or measured RMR values have been used in bioenergetics models [63–67]. Results vary greatly among models with varying assumptions, and the largest variation is generally caused by uncertainty in energy requirements and diet [64]. The ingestion rates for animals under human care have been used to provide estimates of energy requirements, but these studies are likely to overestimate metabolic rates (see references in [50]). A number of studies have measured RMR in dolphins under human care [17–21,68], but the current study is the first to provide estimates for wild animals. Our results indicate that RMR overlaps (RMR in current study for all animals: 3.7 ± 1.8 ml O_2_ min^−1^ kg^−1^, range: 0.8–9.4 ml O_2_ min^−1^ kg^−1^) with those in previous studies for animals under human care (3.0–7.4 ml O_2_ min^−1^ kg^−1^) [17–19,21]. These results are similar to the values estimated by Kleiber's equation and provide a measured value that can be used in bioenergetics models for dolphins [69]. As RMR did not vary with *M*_b_ (range: 141–291 kg) in adult dolphins, the average $\dot{\text{V}}{\text{O}}_{2}$ (592 ml O_2_ min^−1^) can be used for an estimate of the RMR for this *M*_b_ range. For a 150 kg dolphin under the seasonal water temperature conditions of our study (spring in Sarasota Bay, FL), the RMR would be 3.9 ml O_2_ min^−1^ kg^−1^. Assuming a multiplier of 3–6 for the daily metabolic rate of active animals [63,70], the metabolic rate would range from 11.7 to 23.4 ml O_2_ min^−1^ kg^−1^ (126–252 MJ kg^−1^ yr^−1^). This value is similar to the field metabolic rates estimated from a bioenergetics model in dolphins [63].

## 5. Conclusion

Numerous studies have tried to identify the energetic requirements of marine mammals, and recently interest as to how anthropogenic disturbances may alter these requirements has increased. For models that attempt to predict how disturbances may alter population levels, an understanding of the eco-physiology of a study species is crucial. These data are important for helping ecologists understand the flow of energy between different trophic levels. However, there is limited understanding about the physiology of marine mammals and how physiological constraints limit survival. The data presented in this paper provide estimates on the energy requirements and respiratory physiology in two age classes (3–34 years of age) of briefly restrained wild bottlenose dolphins that had been free-ranging just prior to measurement. These data will help improve estimates from bioenergetics models and contribute to our understanding of how a changing environment may alter survival in this species.

## Data accessibility

The model and datasets used in this study are freely available at the following link: https://osf.io/6wjh8/ [71].
